# Facile Construction of Carboxyl-Functionalized Ionic Polymer towards Synergistic Catalytic Cycloaddition of Carbon Dioxide into Cyclic Carbonates

**DOI:** 10.3390/ijms231810879

**Published:** 2022-09-17

**Authors:** Ying Chen, Yingjun Li, Hu Wang, Zaifei Chen, Yi-Zhu Lei

**Affiliations:** 1 College of Environmental and Chemical Engineering, Dalian University, Dalian 116622, China; 2 Guizhou Provincial Key Laboratory of Coal Clean Utilization, School of Chemistry and Materials Engineering, Liupanshui Normal University, Liupanshui 553004, China

**Keywords:** CO_2_ cycloaddition, cyclic carbonates, bifunctional catalyst, ionic polymers

## Abstract

The development of bifunctional ionic polymers as heterogeneous catalysts for effective, cocatalyst- and metal-free cycloaddition of carbon dioxide into cyclic carbonates has attracted increasing attention. However, facile fabrication of such polymers having high numbers of ionic active sites, suitable types of hydrogen bond donors (HBDs), and controlled spatial positions of dual active sites remains a challenging task. Herein, imidazolium-based ionic polymers with hydroxyl/carboxyl groups and high ionic density were facilely prepared by a one-pot quaternization reaction. Catalytic evaluation demonstrated that the presence of HBDs (hydroxyl or carboxyl) could enhance the catalytic activities of ionic polymers significantly toward the CO_2_ cycloaddition reaction. Among the prepared catalysts, carboxyl-functionalized ionic polymer (PIMBr-COOH) displayed the highest catalytic activity (94% yield) in the benchmark cycloaddition reaction of CO_2_ and epichlorohydrin, which was higher than hydroxyl-functionalized ionic polymer (PIMBr-OH, 76% yield), and far exceeded ionic polymer without HBDs groups (PIMBr, 54% yield). Furthermore, PIMBr-COOH demonstrated good recyclability and wide substrate tolerance. Under ambient CO_2_ pressure, a number of epoxides were smoothly cycloadded into cyclic carbonates. Additionally, density functional theory (DFT) calculation verified the formation of strong hydrogen bonds between epoxide and the HBDs of ionic polymers. Furthermore, a possible mechanism was proposed based on the synergistic effect between carboxyl and Br^−^ functionalities. Thus, a facile, one-pot synthetic strategy for the construction of bifunctional ionic polymers was developed for CO_2_ fixation.

## 1. Introduction

Exploring efficient technologies to reduce carbon emission has become the focus of international concern due to the ever-increasing atmospheric CO_2_ concentration [1,2]. Despite the difficulties, the utilization of CO_2_ as a renewable building block for the production of value-added chemicals is deemed one of the most hopeful strategies [3,4]. Recently, numerous transformation approaches have been developed for CO_2_ fixation [5,6]. As a result, catalytic conversion of CO_2_ into valuable cyclic carbonates has attracted enormous attention owing to 100% atom efficiency and high product selectivity [7,8,9].

For chemical conversion of the inert CO_2_ molecules, effective catalysts are essential to overcome the large thermodynamic energy barrier. In this context, various catalytic systems, such as metal salts [10,11,12], imidazolium salts [13,14,15], and ammonium salts [16,17,18], have been developed for CO_2_ cycloaddition during the past few decades [19,20]. Generally, metal-based catalysis systems display high catalytic activity, but they also suffer from obvious shortcomings, such as the contamination of metal residue and difficulty of product separation [21,22,23]. One plausible approach to address these issues is to develop highly active metal-free catalytic systems [24,25,26]. Recent advances have demonstrated that organic halide salts tailoring suitable HBDs could enhance the catalytic activity significantly, thus providing a promising strategy for narrowing the gap between metal-free and metallic catalytic systems [27,28]. Therefore, a number of HBDs-derived homogeneous catalysts have been developed for CO_2_ cycloaddition in recent years [29,30,31,32]. Despite the significant advances, homogeneous catalysts are still restrained due to their inherent disadvantages, such as the high cost of product separation and the difficulty of catalyst recycling.

Poly(ionic liquids) (PILs), which represent a new class of charged polymers featuring high ionic density and excellent CO_2_-affinity, have shown enormous potential as heterogeneous catalysts for CO_2_ fixation [33,34]. During the past decade, numerous PILs with Br^−^ as counterions have been fabricated for cocatalyst and metal-free cycloaddition of CO_2_ into cyclic carbonate, owing to the fact that Br^−^ can promote this conversion via nucleophilicity, attacking the β-carbon atom with little steric hindrance in the epoxide ring [13,35,36]. Nevertheless, PILs with monofunctional Br^−^ generally exhibit inferior catalytic activity. To address this problem, a number of PILs bearing HBDs as electrophile nucleophiles and Br^−^ as nucleophiles have been developed. Previous studies have shown that PILs functionalized with HBDs, such as hydroxyl [37,38,39,40], carboxyl [41,42,43], and amino [44,45], exhibit enhanced catalytic performance, thus showing great potential as promising heterogeneous catalysts for CO_2_ cycloaddition [46]. However, facile fabrication of bifunctional PILs with high numbers of ionic active sites, adjustable types of HBDs, and controlled spatial positions of dual active sites still remains a challenging task.

Herein, HBDs-functionalized ionic polymers were successfully constructed based on the polymerization of commercially available 1,3,5-tri(1H-imidazol-1-yl)benzene and 1,3-dibromo compounds using a one-pot quaternization strategy. Through this facile method, bifunctional PILs bearing high numbers of imidazolium ionic sites, different types of HBDs, and definite spatial positions of HBDs near Br^−^ anions were successfully prepared. Consequently, the as-prepared ionic polymer with a carboxyl group (PIMBr-COOH) showed high catalytic efficiency and nice reusability toward CO_2_ cycloaddition. On the basis of the experimental results and DFT calculation, a possible mechanism involving the synergistic effect of carboxyl and Br^−^ functionalities was proposed.

## 2. Results and Discussion

### 2.1. Preparation and Characterization of the Polymers

**Scheme 1:**
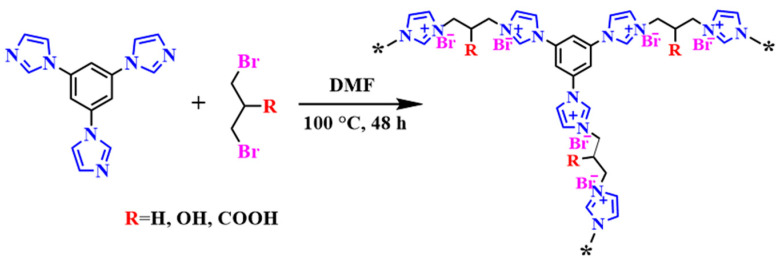
Synthetic scheme of PIMBr, PIMBr−OH, and PIMBr−COOH(* means repeating units of the polymer).

As depicted in Scheme 1, imidazolium-based ionic polymers bearing hydroxyl and carboxyl groups, namely PIMBr-OH and PIMBr-COOH, were facilely fabricated via the quaternization of commercially available 1,3,5-Tri-(1H-imidazol-1-yl) benzene (TIB) with 1,3-dibromo-2-propanol and 3-bromo-2-(bromomethyl)propanoic acid, respectively. For comparison, HBDs-free ionic polymer (PIMBr) was also prepared through the reaction of TIB and 1,3-dibromopropane. As hypercrosslinked polymers, the as-obtained PIMBr, PIMBr-OH, and PIMBr-COOH were insoluble in common solvents (DMF, DMSO, acetonitrile, ethanol, water, THF, etc.).

**Figure 1:**
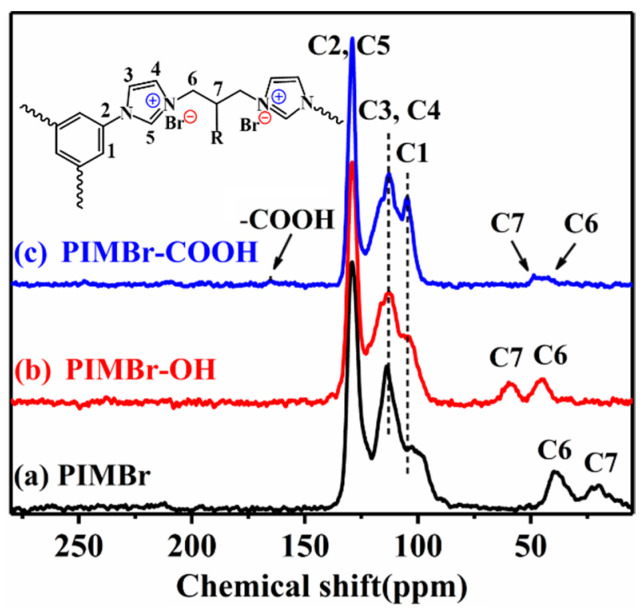
Solid-state ^13^C NMR spectra of (**a**) PIMBr, (**b**) PIMBr−OH, and (**c**) PIMBr−COOH.

To investigate the physicochemical properties of the prepared ionic polymers, a number of characterization analyses, including solid-state ^13^C NMR spectra, FT-IR, XPS, TEM, SEM, SEM-mapping, N_2_ adsorption–desorption, and TGA, were carried out. As seen in Figure 1, solid-state ^13^C NMR spectra of PIMBr, PIMBr-OH, and PIMBr-COOH showed similar signal patterns, suggesting their similar chemical structure. The characteristic peaks at about 128, 113, and 105 ppm were attributable to C2 and C5, C3–4, and C1 atoms of the polymer molecules [34,35]. In the spectrum of PIMBr, the weak signals at around 42 and 47 ppm corresponded to C6–7 atoms of the saturated carbon [38]. Compared to the spectrum of PIMBr, the bands of C6–7 atoms in PIMBr-OH and PIMBr-COOH showed obvious blue shifts due to the presence of -OH and -COOH groups [38]. It was noteworthy that PIMBr-COOH showed a characteristic peak at around 165 ppm, which was related to the carbon atoms of the carboxyl group, thus suggesting the successful incorporation of the carboxyl group in PIMBr-COOH [47,48].

**Figure 2:**
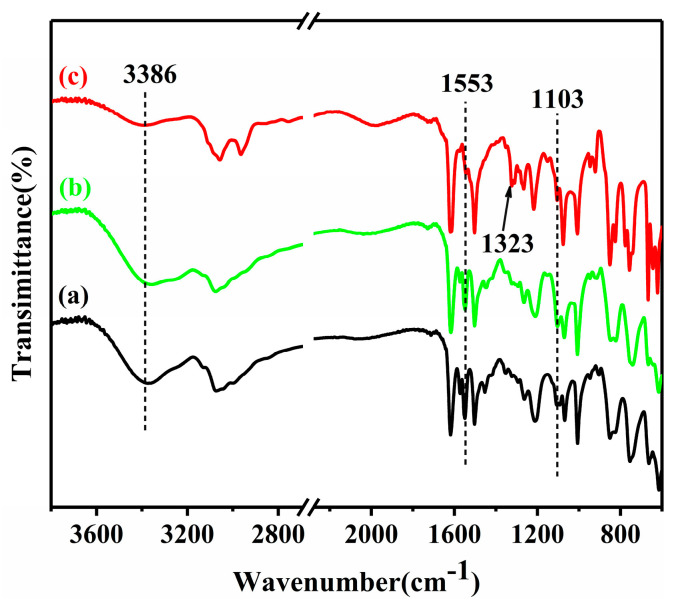
FT-IR spectra of (**a**) PIMBr, (**b**) PIMBr−OH, and (**c**) PIMBr−COOH.

The FT-IR spectra of the three samples in Figure 2 displayed characteristic peaks of imidazolium rings at 1553 and 1105 cm^−^^1^, which were attributed to the stretching vibrations of C = C [49,50] and C-N^+^ bonds [51], respectively. These observations suggested the successful incorporation of the imidazolium units in the prepared polymers. For the spectrum of PIMBr-COOH, the characteristic peak depicted at 1323 cm^−^^1^ was related to the C-O vibration of -COOH groups [52], indicating the successful incorporation of the carboxyl group in PIMBr-COOH. The bands at around 3386 cm^−^^1^ presented in the three samples were attributed to the peak of the absorbed H_2_O. Indeed, the adsorption of atmospheric H_2_O was a common phenomenon in PILs due to their hygroscopic properties [38,53]. Thus, FT-IR analyses further proved the successful fabrication of target ionic polymers.

**Figure 3:**
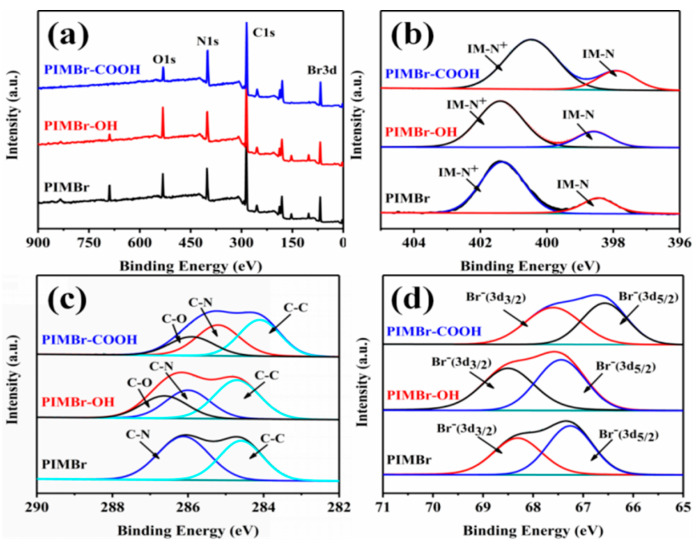
XPS spectra of PIMBr, PIMBr−OH, and PIMBr−COOH. (**a**) Survey scan XPS, (**b**) N 1s, (**c**) C 1s, and (**d**) Br 3d.

**Figure 4:**
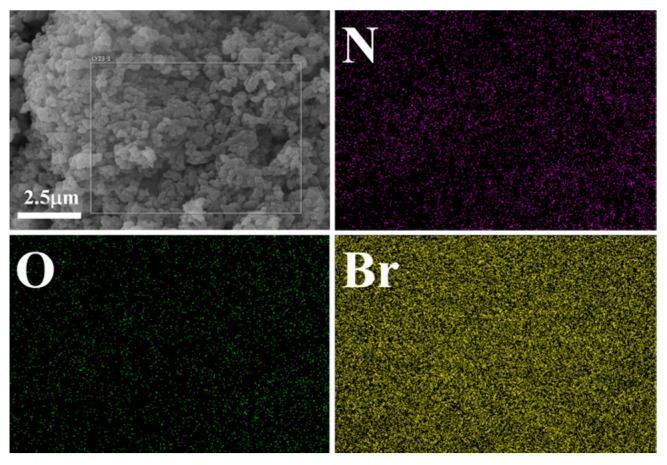
EDS Mapping images of PIMBr-COOH.

XPS was conducted to further analyze the chemical structure of the prepared polymers. As depicted in Figure 3a, C, N, O, and Br elements were identified in the XPS full spectra of PIMBr, PIMBr-OH, and PIMBr-COOH. The presence of the O element in PIMBr could be attributed to the absorbed H_2_O in the polymer network, which was consistent with the result of the FT-IR analysis. As seen in Figure 3b, the N 1s spectra exhibited two different peaks at 401 eV and 397 eV, corresponding to the contribution of ionic and non-ionic N atoms of the imidazolium rings, respectively [54,55,56]. The C 1s spectra of PIMBr-OH and PIMBr-COOH in Figure 3c can be deconvoluted into three peaks at around 286, 285, and 284 eV, which were attributable to C-O, C-N, and C-C bonds, respectively [38,54,57]. The Br 3d spectra in Figure 3d were fitted into two peaks related to the 3d_3/2_ and 3d_5/2_ binding energy of Br^−^ [58]. Therefore, XPS analyses further demonstrated the successful fabrication of target polymers. Element distribution of PIMBr-COOH was also investigated by EDS mapping. As illustrated in Figure 4, N, O, and Br elements were homogeneously distributed in the ionic polymer framework, suggesting that the active sites of the prepared catalyst were well-distributed. For solid catalysts bearing synergistic catalytic sites, the homogeneous distribution of the active sites was important for achieving synergistic catalytic performance.

The porous properties of the prepared polymers were measured by nitrogen physisorption at 77 K. As summarized in Table 1, PIMBr and PIMBr-OH showed low specific surface areas of 8.47 and 11.20 m^2^ g^−1^, respectively, which were similar to those of ionic polymers synthesized by the quaternization reaction [59,60]. PIMBr-COOH revealed a moderate surface area of 36.51 m^2^ g^−1^ that was higher than that of PIMBr-OH. The relatively low surface areas of the three polymers could be ascribed to their high ionic density and flexible propylene linkers. High ionic density led to strong electrostatic interactions between the polymer chains [59,60], while the flexibility of the propylene linkers resulted in framework interpenetration during the framework formation.

**Figure 5:**
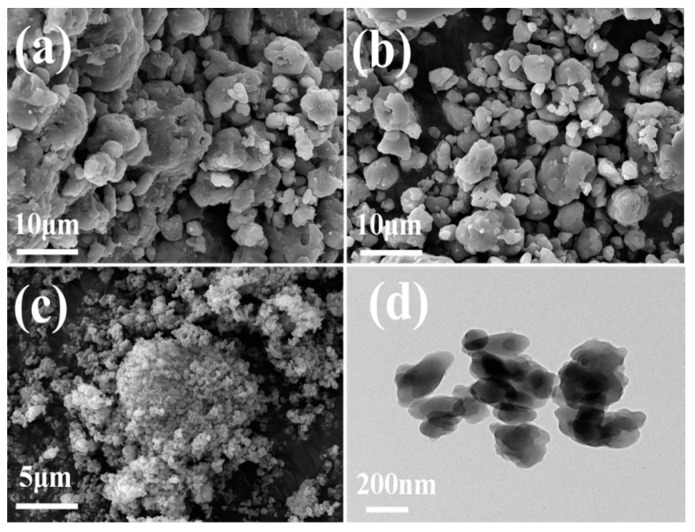
SEM images of (**a**) PIMBr, (**b**) PIMBr-OH, and (**c**) PIMBr-COOH. (**d**) TEM image of PIMBr-COOH.

The morphologies of the prepared polymers were analyzed SEM (Figure 5). It is worth noting that PIMBr-COOH showed a sponge-like morphology that comprised irregular reunion particles. This rough surface structure endowed the polymer with abundant absorption and active sites for CO_2_ fixation. PIMBr and PIMBr-OH displayed irregular agglomeration structures of large particles, and the particle size was much greater than that of PIMBr-COOH. A representative TEM image of PIMBr-COOH (Figure 5d) revealed that the diameter of the aggregated particles was in a range of tens to hundreds of nanometers.

**Figure 6:**
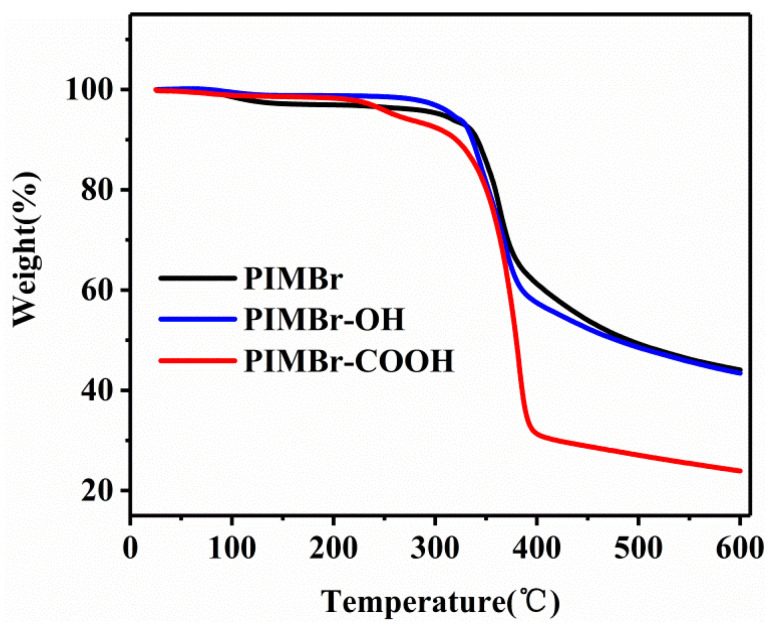
TGA curves for PIMBr, PIMBr-OH, and PIMBr-COOH.

As polymer-based solid catalysts, the thermal stability of the polymers is a critical aspect of their practical application. Therefore, TGA was conducted to analyze their thermal stability over the temperature range of 25–600 °C. As depicted in Figure 6, three samples showed similar thermogravimetric behavior. The initial weight loss (2–5 wt%) that occurred below 150 °C could be assigned to the removal of the absorbed water, a common phenomenon for ionic polymers [49,61]. For PIMBr and PIMBr-OH, the main weight loss took place above 250 °C. Compared to PIMBr and PIMBr-OH, PIMBr-COOH exhibited a lower decomposition temperature and a higher weight loss rate. These could be owing to the high oxygen content and easy decomposition of –COOH groups. TGA results indicated that the three samples could be stable at 220 °C. For CO_2_ cycloaddition, the reaction was generally performed below 150 °C; therefore, the prepared polymers were stable enough to promote such CO_2_ conversion.

### 2.2. Catalytic Performance

**Figure 7:**
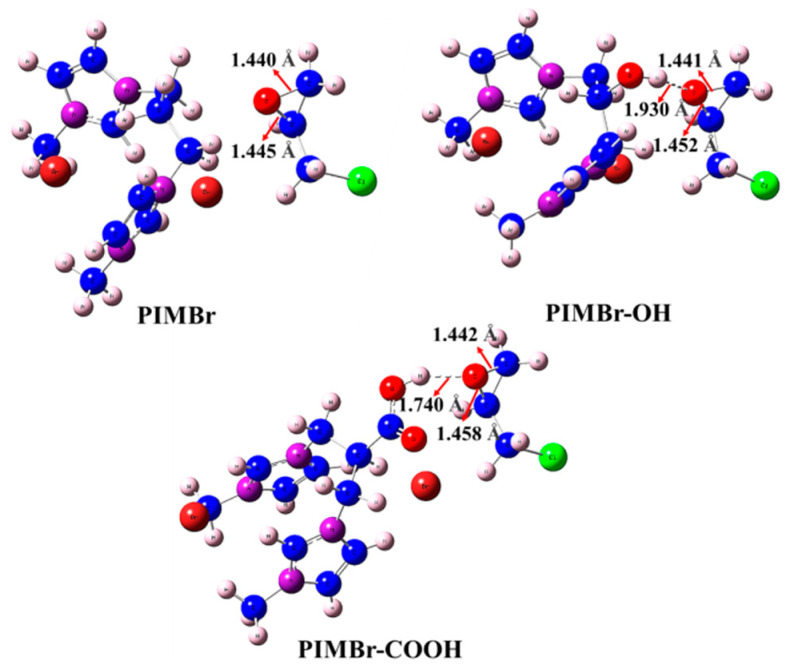
DFT optimized geometry of the interaction between epichlorohydrin and PILs.

With the target ionic polymers in hand, their catalytic performance was evaluated in the CO_2_ cycloaddition reaction to certify the rational design of HBDs-functionalized PILs. At the beginning of the study, catalytic activities of PIMBr, PIMBr-OH, and PIMBr-COOH were compared in the cycloaddition of CO_2_ into less-volatile epichlorohydrin (model reaction). As summarized in Table 2, 1 mol% of HBDs-free PIMBr afforded a 54% yield of target cyclic carbonate at 100 °C for 4 h (Table 2, entry 1). Notably, under the same reaction conditions, PIMBr-OH and PIMBr-COOH provided much higher yields, reaching up to 76% and 94%, respectively (Table 2, entries 2 and 3). Given that PIMBr, PIMBr-OH, and PIMBr-COOH had similar chemical structures and ionic densities, the higher catalytic activities of PIMBr-OH and PIMBr-COOH can be due to the synergistic catalytic effect between hydroxyl/carboxyl HBDs and Br^−^ [62,63,64], which was able to promote the rate-determining step (i.e., epoxide ring opening) of the reaction, thereby accelerating the reaction rate. Compared to PIMBr-OH, the higher catalytic activity of PIMBr-COOH should be ascribed to its stronger hydrogen bonding interaction with epichlorohydrin molecular [47]. To confirm these assumptions, DFT calculations were carried out. As depicted in Figure 7, the results of DFT calculation were consistent with those of catalytic evaluation. Hydrogen bonds with the bond lengths of 1.930 and 1.740 Å were formed between epichlorohydrin, PIMBr-OH, and PIMBr-COOH. The shorter H-bond length between epichlorohydrin and PIMBr-COOH suggested a stronger hydrogen bond interaction. Accordingly, the bond length of the C–O bond in the epichlorohydrin ring was lengthened from 1.440 Å to 1.441 and 1.442 Å after interaction with PIMBr-OH and PIMBr-COOH, respectively. Therefore, the results of the catalytic evaluation and DFT calculations verified the importance of suitable HBDs in boosting the catalytic efficiency of ionic polymers.

**Figure 8:**
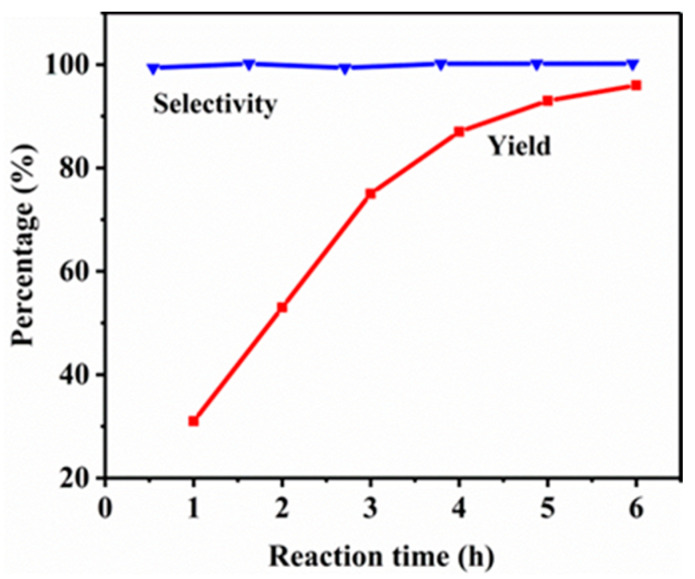
Effect of reaction time. Epichlorohydrin (43 mmol), PIMBr-COOH (1 mol%, 90 mg), 100 °C, and CO_2_ pressure (0.1 MPa).

Having the optimal catalyst in hand, the influences of reaction parameters, such as temperatures, CO_2_ pressure, and reaction time, were investigated to optimize the reaction conditions. As illustrated in Table 3, the results indicate that reaction temperature had a great impact on the catalytic activity. The yield of target cyclic carbonate exhibited an obvious increment when the reaction temperature increased from 40 to 100 °C (Table 3, entries 1–3). At 100 °C, the effect of CO_2_ pressure was also evaluated. As summarized in Table 3, the product yield decreased gradually as the CO_2_ pressure reduced from 1 MPa to 0.1 MPa. Notably, 77% yield and high selectivity of target carbonate were still observed at ambient CO_2_ pressure. The good catalytic performance under low CO_2_ pressure should be assigned to the high ionic density and porous structure of the PIMBr-COOH catalyst. Under ambient CO_2_ pressure, the effect of reaction time was also checked. As displayed in Figure 8, the product yield increased from 31% to 96% when the reaction time extended from 1 to 6 h. Therefore, we obtained the optimal reaction conditions: PIMBr-COOH (1 mol%), 100 °C, CO_2_ pressure (0.1 MPa), and 6 h.

**Figure 9:**
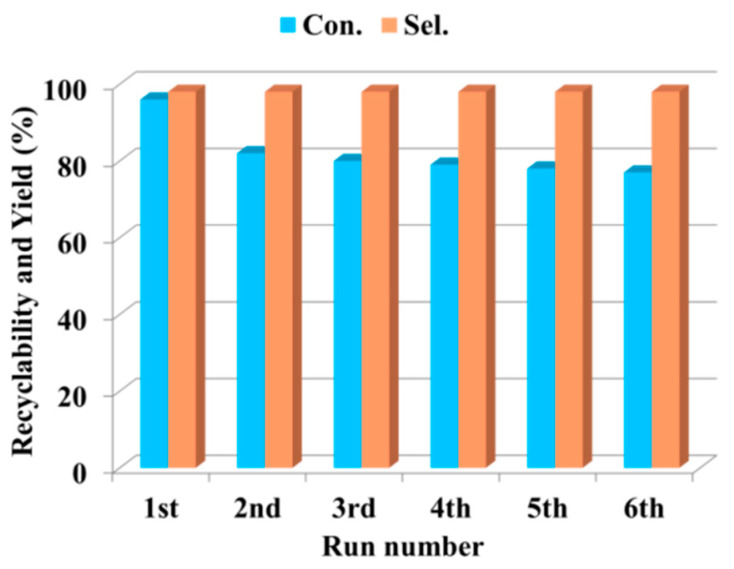
Catalytic reusability of PIMBr-COOH. Reaction conditions: epichlorohydrin (43 mmol), CO_2_ pressure (0.1 MPa), the catalyst PIMBr-COOH (90 mg, 1 mol%), 100 °C, and 6 h.

Apart from catalytic activity, the recyclability of the solid catalysts is also a crucial indicator for evaluating their potential in industrial applications. Therefore, the recycling performance of PIMBr-COOH was evaluated under optimal reaction conditions. At the end of each catalytic run, the solid catalyst was centrifugally separated, washed with ethanol, dried under a vacuum, and then directly engaged in the next catalytic cycle. As shown in Figure 9, PIMBr-COOH showed nice recyclability and could be used at least six times. The initial activity decrease after the first run might be attributed to the partial pore blocking during the first catalytic run. For comparison, the catalytic performance of some previously reported metal and cocatalyst-free heterogeneous catalysts is listed in Table 4. Notably, PIMBr-COOH showed better catalytic performance than most of the previously reported solid and metal-free catalysts. In terms of the salient features, such as facile preparation, high numbers of ionic active sites, controlled spatial positions of dual active sites, high catalytic activity, and effective catalyst recyclability, the designed bifunctional ionic polymer was shown to be a promising heterogeneous and environmentally friendly catalyst for CO_2_ cycloaddition.

To investigate the general applicability of the PIMBr-COOH catalyst in the CO_2_ cycloaddition reaction, epoxides bearing different functional groups were tested. As shown in Table 5, epoxides with electron-withdrawing groups, such as bromide and alkoxy, worked well and reacted with CO_2_ smoothly to afford excellent yields of corresponding products in 6 h (Table 5, entries 1–4). Epoxides bearing electron-donating alkyl groups could also provide excellent yields of target cyclic carbonates, but prolonged reaction times are needed (Table 5, entries 5 and 6). As a representative example of sterically hindered epoxide, styrene oxide provided a high yield of corresponding cyclic carbonate in 6 h (Table 5, entry 7). These results further revealed that the prepared PIMBr-COOH is a promising heterogeneous catalyst for CO_2_ cycloaddition reaction.

### 2.3. Plausible Reaction Mechanism

**Scheme 2:**
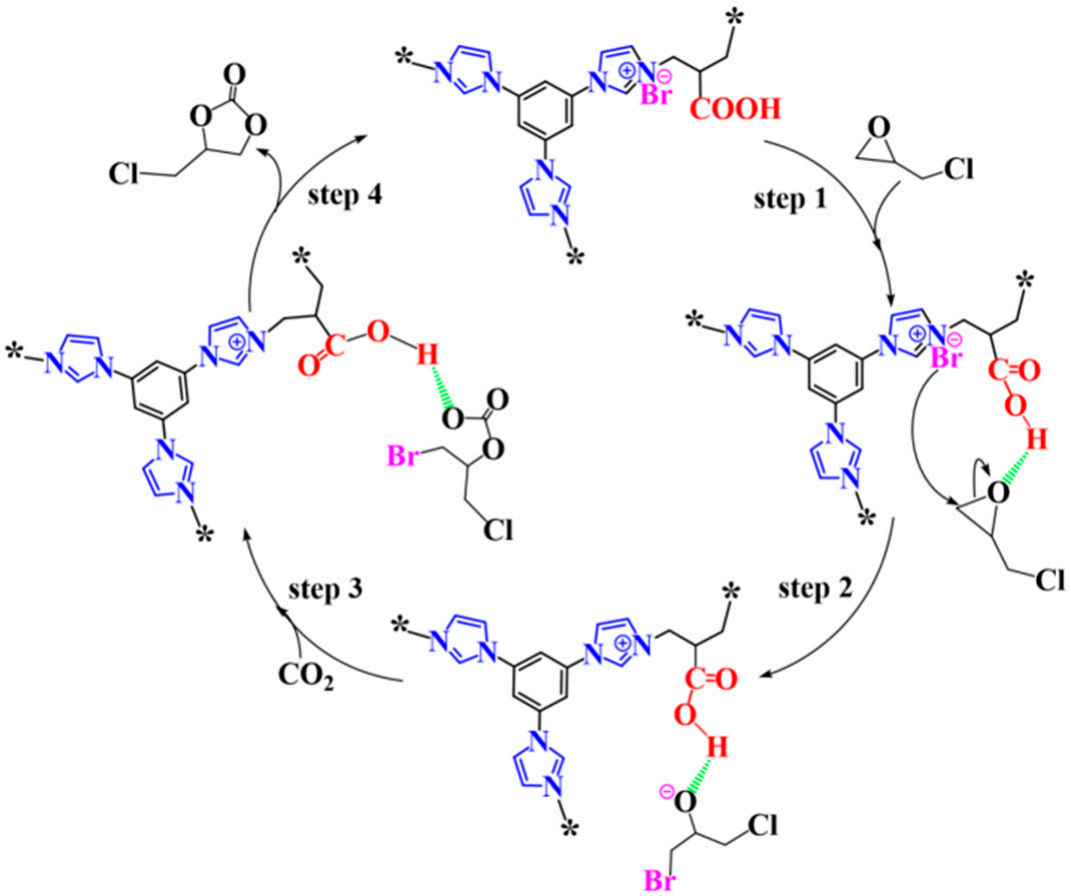
Possible catalytic mechanism for the cycloaddition of CO_2_ with epichlorohydrin catalyzed by PIMBr-COOH(* means repeating units of the polymer).

Based on the experimental results and literature data [44,48,49,70], a plausible mechanism for CO_2_ cycloaddition reaction using PIMBr-COOH as a catalyst was proposed (Scheme 2). Firstly, the carboxyl group in PIMBr-COOH coordinated with the oxygen atom in epichlorohydrin, leading to the formation of a hydrogen bond and thereby activating the C-O bond of the epichlorohydrin ring. Subsequently, the epichlorohydrin ring was opened via the nucleophilic attack of Br^−^ on the β-carbon atom with little steric hindrance in epichlorohydrin, leading to the formation of a bromoalkoxide intermediate. Then, an alkylcarbonate anion was produced through the reaction between the bromoalkoxide intermediate and CO_2_. Lastly, target cyclic carbonate was generated via the intermolecular ring-closing of the alkylcarbonate anion. Meanwhile, PIMBr-COOH was regenerated and participated in the next catalytic cycle.

## 3. Methods and Materials

### 3.1. Chemicals

1,3-Dibromopropane (98%), *N,N*-dimethylformamide (99.9%), ethanol, 3-bromo-2-(bromomethyl)propionic acid (98%), 1,3-dibromo-2-propanol (95%), and epoxides were of analyzed grades and purchased from Energy Chemical (Shanghai, China). TIB (98%) was obtained from Alchemy Co. Ltd. CO_2_ was obtained from a local manufacturer with a purity of 99.9%.

### 3.2. Synthesis of PIMBr, PIMBr-OH and PIMBr-COOH

HBDs-functionalized PILs were fabricated via a one-pot quaternization strategy. In detail, TIB (1.73 mmol, 0.48 g) and 3-bromo-2-(bromomethyl)propionic acid (2.32 mmol, 0.57 g) were added to *N,N*-dimethylformamide (30 mL) in a 100 mL pressure-resistant tube. After replacing the interior air with N_2_, the tube was transferred to an oil bath, stirred, and heated under 100 °C for 24 h. Subsequently, the tube was removed from the oil bath and cooled naturally to room temperature. Next, the resultant solid was washed thoroughly with ethanol, filtered, and dried under vacuum (80 °C) for 24 h. Finally, 1.03 g of white solid (98% yield) were obtained and named PIMBr-COOH (1.03 g). Following the same synthesis procedures of PIMBr-COOH, PIMBr and PIMBr-OH were also successfully fabricated in nearly quantitative yields by taking the place of 3-bromo-2-(bromomethyl)propionic acid with other 1,3-dibromo compounds, i.e., 1,3-dibromopropane and 1,3-dibromo-2-propanol, respectively.

### 3.3. Catalytic Performance

In a typical reaction of catalytic CO_2_ cycloaddition, catalysts (PIMBr-COOH, PIMBr-OH, or PIMBr) and epoxide were successively added to a 25 mL high-pressure reactor equipped with a magnetic stirrer. After purging with CO_2_ several times, the reactor was charged with CO_2_ to 1 MPa and reacted at a pre-set temperature for the required time. After reaction completion, the system was cooled to room temperature. The resultant mixture was separated by centrifugation, and the obtained liquid was analyzed by GC and GC-MS using dimethylacetamide as the internal standard.

### 3.4. Characterization

Solid-state ^13^C NMR measurements were analyzed on a Bruker Avance 400 NMR spectrometer (Zurich, Switzerland) with a 4 mm rotor spinning module. FT-IR measurements were recorded on a Nicolet Nexus FT-IR spectrometer (Madison, WI, USA) using the KBr pellet technique over a range of 4000–400 cm^−1^ at 4 cm^−^^1^ resolution and 32 scans. X-ray photoelectron spectroscopy (XPS) analyses of the prepared PILs were performed on a Thermo Fisher Scientific K-Alpha spectrometer (Waltham, MA, USA) equipped with Al K radiation (1486.68 eV). Porous properties of the prepared PILs were measured at 77 K on a Micromeritics ASAP2460 after degassing at 100 °C for 8 h. A scanning electron microscope (FE-SEM, Hitachi S-4800, accelerateel voltage: 5 kV (Tokyo, Japan) with energy-dispersive X-ray spectroscopy (EDS) and a transmission electron microscope (TEM, FEI Talos F200X), operating at a high voltage of 200 kV, were used to investigate the morphology of the materials. Thermogravimetric analysis (TGA, Netzsch, Bavaria, Germany) was carried out using a NETZSCH STA 449F3 over a temperature range from 25 to 600 °C at a heating rate of 10 °C/min under a dynamic N_2_ atmosphere. GC and GC-MS analyses were recorded on SCION 456-GC and Agilent 6890/5973 GC-MS, respectively.

### 3.5. DFT Calculation

DFT calculations were performed to investigate the hydrogen-bond interactions between epichlorohydrin and designed catalysts using the Gaussian 09 package. The geometry of all molecules was optimized by the B3LYP-GD3 level using the 6-31G* [25] basis set [71]. The same level of frequency analysis was used to ensure the minimum potential energy surface of the optimized geometry.

## 4. Conclusions

In summary, novel ionic polymers with different types of HBDs, high numbers of imidazolium ionic sites, and controlled spatial positions of dual active sites were facilely constructed by a one-pot quaternization strategy. Impressively, the presence of hydroxyl and carboxyl groups boosted the catalytic activities of ionic polymers significantly toward the cycloaddition reaction of CO_2_ into epoxides. Carboxyl-functionalized ionic polymers displayed higher catalytic activity than those of HBDs-free and hydroxyl-functionalized ionic polymers. DFT calculation demonstrated that the formation of hydrogen bonds between epoxide with HBDs of ionic polymers facilitated the ring-opening step, and the higher catalytic activity of PIMBr-COOH was mainly attributed to its stronger hydrogen bond interaction with the epoxide substrate. Additionally, PIMBr-COOH exhibited good recyclability and was able to be reused at least five times. Under ambient CO_2_ presence, a series of substituted epoxides were smoothly transformed into cyclic carbonates in excellent yields under solvent-, cocatalyst-, and metal-free reaction conditions. This research thus represents a reliable strategy for direct fabrication of carboxyl-functionalized ionic polymers towards efficient CO_2_ conversion.

## Figures and Tables

**Table 1:** Physical properties of PIMBr, PIMBr-OH, and PIMBr-COOH.

Entry	Samples	S_BET_ (m^2^·g^−1^) ^a^	V (cm^3^ g^−1^) ^b^	D (nm) ^c^
1	PIMBr	8.47	0.14	65.69
2	PIMBr-OH	11.20	0.15	54.89
3	PIMBr-COOH	36.51	0.28	30.90

^a^ BET surface area. ^b^ Total pore volume. ^c^ Adsorption average pore diameter (4 V/A by BET).

**Table 2:** Catalytic performances of different catalysts in the cycloaddition of epichlorohydrin with CO_2_ ^a^.

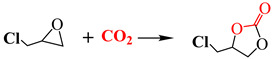			
Entry	Catalysts	Yield (mol%) ^b^	Sel. (mol%) ^c^
1	PIMBr	54	>99
2	PIMBr-OH	76	>99
3	PIMBr-COOH	94	>99

^a^ Reaction conditions: epichlorohydrin (43 mmol), catalyst (1 mol%, according to the amount of Br^−^), CO_2_ (1 MPa), reaction temperature (100 °C), and 4 h. ^b^ Determined by GC. ^c^ 3-Chloro-1,2-propanediol was identified as the only by-product.

**Table 3:** Effect of temperature and CO_2_ pressure for the cycloaddition of epichlorohydrin with CO_2_^a^.

Entry	Temp. (°C)	PCO_2_ (MPa)	Yield (mol%) ^b^	Sel. (mol%) ^c^
1	60	1	4	>99
2	80	1	44	>99
3	100	1	94	>99
4	100	0.5	90	>99
5	100	0.25	82	>99
6	100	0.1	77	>99

^a^ Reaction conditions: epichlorohydrin (43 mmol), PIMBr-COOH (1 mol%, 90 mg), 100 °C, and 4 h. ^b^ Determined by GC. ^c^ 3-Chloro-1,2-propanediol was identified as the only by-product.

**Table 4:** Comparison of the catalytic performance of PIMBr-COOH with some previously reported metal and cocatalyst-free heterogeneous catalysts under close reaction conditions.

Entry	Catalysts	Epoxide ^a^	Catal. Usage ^b^	Conditions	Yield (mol%)	Reused Times	Ref.
1	PIMBr-COOH	ECH	1 mol%	100 °C, CO_2_ 0.1 MPa, 6 h	96	5	This work
2	PCm_3_Lm1Nm_2_	PO	2 mol%	100 °C, CO_2_ 1.2 MPa, 8 h.	79	4	[65]
3	PDMBr	PO	1.3 mol%	110 °C, CO_2_ 1 MPa, 4 h	98.7	5	[66]
4	SAIL-4	PO	3 mol%	100 °C, CO_2_ 1.5 MPa, 3 h	99	5	[67]
5	PQPBrCOOH	ECH	1 mol%	120 °C, CO_2_ 0.1 MPa, 8 h	97.8	8	[25]
6	PIL-2	ECH	10 mg	100 °C, CO_2_ 1 MPa, 4 h	81.2	-	[17]
7	CTF-TPM-400	ECH	3 wt%	100 °C, CO_2_ 0.7 MPa, 24 h	99	5	[68]
8	ImIP@TT-COF	ECH	~3.52 wt%	120 °C, CO_2_ 0.1 MPa, 10 h	99	6	[69]

^a^ Epichlorohydrin, ECH; propylene epoxide, PO. ^b^ Based on the amount of Br^−^.

**Table 5:** Cycloaddition of CO_2_ with various epoxides catalyzed by PIMBr-COOH ^a^.

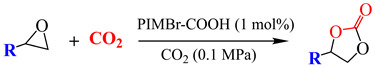				
Entry	R	T (h)	Yield (mol%) ^b^	Sel. (mol%)
1	-CH_2_Br	6	96	>99
2	-CH_2_O(CH_2_)_3_CH_3_	6	95	>99
3	-CH_2_OPh	6	98	>99
4	-CH_2_OCH_2_Ph	6	94	>99
5	-CH_3_	10	99	>99
6	-(CH_2_)_3_CH_3_	10	99	>99
7	-Ph	6	90	>99

^a^ Reaction conditions: epoxide (43 mmol), PIMBr-COOH (1 mol%, 90 mg), CO_2_ (0.1 MPa), and 100 °C. ^b^ Determined by GC.

## Data Availability

Not applicable.

## References

[1] Zhang Z.E., Pan S.Y., Li H., Cai J.C., Olabi A.G., Anthony E.J., Manovic V. (2020). Recent advances in carbon dioxide utilization. Chem. Rev..

[2] Artz J., Müller T.E., Thenert K., Kleinekorte J., Meys R., Sternberg A., Bardow A., Leitner W. (2018). Sustainable conversion of carbon dioxide: An integrated review of catalysis and life cycle assessment. Chem. Rev..

[3] Truong C.C., Mishra D.K. (2020). Recent advances in the catalytic fixation of carbon dioxide to value-added chemicals over alkali metal salts. J. CO_2_ Util..

[4] Yaashikaa P.R., Kumar P.S., Varjani S.J., Saravanan A. (2019). A review on photochemical, biochemical and electrochemical transformation of CO_2_ into value-added products. J. CO_2_ Util..

[5] Huang K., Zhang J.Y., Liu F., Dai S. (2018). Synthesis of porous polymeric catalysts for the conversion of carbon dioxide. ACS Catal..

[6] Rehman A., Nazir G., Rhee K.Y., Park S.J. (2022). Electrocatalytic and photocatalytic sustainable conversion of carbon dioxide to value-added chemicals: State-of-the-art progress, challenges, and future directions. J. Environ. Chem. Eng..

[7] Aomchad V., Cristòfol A., Monica F.D., Limburg B., D’Elia V., Kleij A.W. (2021). Recent progress in the catalytic transformation of carbon dioxide into biosourced organic carbonates. Green Chem..

[8] Pescarmona P.P. (2021). Cyclic carbonates synthesised from CO_2_: Applications, challenges and recent research trends. Curr. Opin. Green Sust..

[9] Rehman A., Saleem F., Javed F., Ikhlaq A., Ahmad S.W., Harvey A. (2021). Recent advances in the synthesis of cyclic carbonates via CO_2_ cycloaddition to epoxides. J. Environ. Chem. Eng..

[10] Han B.X., Jiang Y.F., Sun X.R., Li Z.F., Li G. (2021). Proton conductive N-heterocyclic metal–organic frameworks. Coord. Chem. Rev..

[11] Zhou H., Wang G.X., Zhang W.Z., Lu X.B. (2015). CO_2_ adducts of phosphorus ylides: Highly active organocatalysts for carbon dioxide transformation. ACS Catal..

[12] Qi C., Chaemchuen S., Liu M., Wang J.C., Zhuiykov S., Verpoort F. (2022). Metal embedded porous carbon for efficient CO_2_ cycloaddition under mild conditions. Catalysts.

[13] Guo L.P., Lamb K.J., North M. (2021). Recent developments in organocatalysed transformations of epoxides and carbon dioxide into cyclic carbonates. Green Chem..

[14] Zhang W.L., Ma F.P., Ma L., Zhou Y., Wang J. (2020). Imidazolium-functionalized ionic hypercrosslinked porous polymers for efficient synthesis of cyclic carbonates from simulated flue gas. ChemSusChem.

[15] Luo R.C., Liu X.Y., Chen M., Liu B.Y., Fang Y.X. (2020). Recent advances on imidazolium-functionalized organic cationic polymers for CO_2_ adsorption and simultaneous conversion into cyclic carbonates. ChemSusChem.

[16] Ebrahimi A., Rezazadeh M., Khosravi H., Rostami A., Al-Harrasi A. (2020). An aminopyridinium ionic liquid: A simple and effective bifunctional organocatalyst for carbonate synthesis from carbon dioxide and epoxides. ChemPlusChem.

[17] Zou Y.Z., Ge Y.S., Zhang Q., Liu W., Li X.G., Cheng G.E., Ke H.Z. (2022). Polyamine-functionalized imidazolyl poly(ionic liquid)s for the efficient conversion of CO_2_ into cyclic carbonates. Catal. Sci. Technol..

[18] Bao C.L., Jiang Y.C., Zhao L.Y., Li D.Z., Xu P., Sun J.M. (2021). Aminoethylimidazole ionic liquid-grafted MIL-101-NH_2_ heterogeneous catalyst for the conversion of CO_2_ and epoxide without solvent and cocatalyst. New J. Chem..

[19] Zhang X., Geng W.H., Yue C.T., Wu W., Xiao L.F. (2016). Multilayered supported ionic liquids bearing a carboxyl group: Highly efficient catalysts for chemical fixation of carbon dioxide. J. Environ. Chem. Eng..

[20] Jiang B.W., Yan X.Y., Xu Y., Likhanova N., Velázquez H.D., Gong Y.Y., Yuan Y., Verpoort F. (2022). Tandem reactions based on the cyclization of carbon dioxide and propargylic alcohols: Derivative applications of α-Alkylidene carbonates. Catalysts.

[21] Zhang Y., Yin S., Luo S., Au C.T. (2012). Cycloaddition of CO_2_ to epoxides catalyzed by carboxyl-functionalized imidazolium-based ionic liquid grafted onto cross-linked polymer. Ind. Eng. Chem. Res..

[22] Zhang N., Zou B., Yang G.P., Yu B., Hu C.W. (2017). Melamine-based mesoporous organic polymers as metal-free heterogeneous catalyst: Effect of hydroxyl on CO_2_ capture and conversion. J. CO_2_ Util..

[23] Ma D.X., Liu K., Li J.X., Shi Z. (2018). Bifunctional metal-free porous organic framework heterogeneous catalyst for efficient CO_2_ conversion under mild and cocatalyst-free conditions. ACS Sustain. Chem. Eng..

[24] Zhang Y.Y., Yang G.W., Xie R., Li L., Yang B., Wu G.P. (2020). Scalable, durable, and recyclable metal-free catalysts for highly efficient conversion of CO_2_ to cyclic carbonates. Angew. Chem. Int. Ed..

[25] Wan Y.L., Zhang Z.M., Ding C., Wen L.L. (2021). Facile construction of bifunctional porous ionic polymers for efficient and metal-free catalytic conversion of CO_2_ into cyclic carbonates. J. CO_2_ Util..

[26] Cokoja M., Wilhelm M.E., Anthofer M.H., Herrmann W.A., Kühn F.E. (2015). Synthesis of cyclic carbonates from epoxides and carbon dioxide by using organocatalysts. ChemSusChem.

[27] Arayachukiat S., Kongtes C., Barthel A., Vummaleti S.V.C., Poater A., Wannakao S., Cavallo L.G., D’Elia V. (2017). Ascorbic acid as a bifunctional hydrogen bond donor for the synthesis of cyclic carbonates from CO_2_ under ambient conditions. ACS Sustain. Chem. Eng..

[28] Liu M.S., Wang X., Jiang Y.C., Sun J.M., Arai M. (2018). Hydrogen bond activation strategy for cyclic carbonates synthesis from epoxides and CO_2_: Current state-of-the art of catalyst development and reaction analysis. Catal. Rev..

[29] Ye Y.F., Li D.Z., Xu P., Sun J.M. (2020). B-doped and NH_2_-functionalized SBA-15 with hydrogen bond donor groups for effective catalysis of CO_2_ cycloaddition to epoxides. Inorg. Chem. Front..

[30] Hu J.Y., Ma J., Liu H.A., Qian Q.L., Xie C., Han B.X. (2018). Dual-ionic liquid system: An efficient catalyst for chemical fixation of CO_2_ to cyclic carbonates under mild conditions. Green Chem..

[31] Chudasama S.J., Shah B.J., Patel K.M., Dhameliya T.M. (2022). The spotlight review on ionic liquids catalyzed synthesis of aza- and oxa-heterocycles reported in 2021. J. Mol. Liq..

[32] Yue S., Wang P.P., Hao X.Y. (2019). Synthesis of cyclic carbonate from CO_2_ and epoxide using bifunctional imidazolium ionic liquid under mild conditions. Fuel.

[33] Zhou X.J., Weber J., Yuan J.Y. (2019). Poly (ionic liquid)s: Platform for CO_2_ capture and catalysis. Curr. Opin. Green Sustain. Chem..

[34] Zhang J.W., Li X.P., Zhu Z., Chang T., Fu X.Y., Hao Y.J., Meng X.C., Panchal B., Qin S.J. (2021). Hydroxylamino—Anchored poly (ionic liquid)s for CO_2_ fixation into cyclic carbonates at mild conditions. Adv. Sustain. Syst..

[35] Wang Y.P., Duan J.X. (2022). Urea and thiourea-functionalized, pyridinium-based ionic polymers convert CO_2_ to cyclic carbonate under mild conditions. ACS Appl. Polym. Mater..

[36] Liao X., Pei B.Y., Ma R.X., Kong L.Z., Gao X.L., He J., Luo X.Y., Lin J.Q. (2022). Hypercrosslinked ionic polymers with high ionic content for efficient conversion of carbon dioxide into cyclic carbonates. Catalysts.

[37] Gou H.B., Ma X.F., Su Q., Liu L., Ying T., Qian W., Dong L., Cheng W.G. (2021). Hydrogen bond donor functionalized poly (ionic liquid)s for efficient synergistic conversion of CO_2_ to cyclic carbonates. Phys. Chem. Chem. Phys..

[38] Zhang Y.D., Chen G.J., Wu L., Liu K., Zhong H., Long Z.Y., Tong M.M., Yang Z.Z., Dai S. (2020). Two-in-one: Construction of hydroxyl and imidazolium- bifunctionalized ionic networks in one-pot toward synergistic catalytic CO_2_ fixation. Chem. Commun..

[39] Liu D., Kang S.M., Xu Y.J., Duan P.G., Chen S.X. (2021). Core-shell catalyst with synergistic hydroxyl and nitrogen active sites for CO_2_ cycloaddition. J. Environ. Chem. Eng..

[40] Zhang Y.W., El-Sayed E.S.M., Su K.Z., Yuan D.Q., Han Z.B. (2020). Facile syntheses of ionic polymers for efficient catalytic conversion of CO_2_ to cyclic carbonates. J. CO_2_ Util..

[41] Wang Y.C., Liu Y.F., Su Q., Li Y.N., Deng L.L., Dong L., Fu M.Q., Liu S.F., Cheng W.G. (2022). Poly (ionic liquid) materials tailored by carboxyl groups for the gas phase-conversion of epoxide and CO_2_ into cyclic carbonates. J. CO_2_ Util..

[42] Yang C.K., Chen Y.L., Wang X., Sun J.M. (2022). Polymeric ionic liquid with carboxyl anchored on mesoporous silica for efficient fixation of carbon dioxide. J. Colloid Interface Sci..

[43] Liu X.F., Song Q.W., Zhang S., He L.N. (2016). Hydrogen bonding-inspired organocatalysts for CO_2_ fixation with epoxides to cyclic carbonates. Catal. Today.

[44] Liu M.S., Zhao P.H., Zhang W.W., Cheng X., Fei H.T., Ma J.J., Liu F.S. (2022). Rational self-assembly of triazine-and urea-functionalized periodic mesoporous organosilicas for efficient CO_2_ adsorption and conversion into cyclic carbonates. Fuel.

[45] Jiang Y.C., Li J.J., Jiang P.P., Li Y., Leng Y. (2018). Amino acid-paired dipyridine polymer as efficient metal-and halogen-free heterogeneous catalysts for cycloaddition of CO_2_ and epoxides into cyclic carbonates. Catal. Commun..

[46] Guo Z.J., Cai X.C., Xie J.Y., Wang X.C., Zhou Y., Wang J. (2016). Hydroxyl-exchanged nanoporous ionic copolymer toward low-temperature cycloaddition of atmospheric carbon cioxide into carbonates. ACS Appl. Mater. Inter..

[47] Wang Y.P., Nie J.Q., Lu C.F., Wang F.Y., Ma C., Chen Z.X., Yang G.C. (2020). Imidazolium-based polymeric ionic liquids for heterogeneous catalytic conversion of CO_2_ into cyclic carbonates. Microp. Mesop. Mater..

[48] Li Y.H., Dominelli B., Reich R.M., Liu B.P., Kühn F.E. (2019). Bridge-functionalized bisimidazolium bromides as catalysts for the conversion of epoxides to cyclic carbonates with CO_2_. Catal. Commun..

[49] Ma Z.Q., Yang Y.Y., Ma Q.Q., Zhou H.Z., Luo X.P., Liu X.H., Wang S.R. (2017). Evolution of the chemical composition, functional group, pore structure and crystallographic structure of bio-char from palm kernel shell pyrolysis under different temperatures. J. Anal. Appl. Pyrol..

[50] Mao P., Dai W.L., Yang W.Y., Luo S.L., Zhang Y., Mao J., Luo X.B., Zou J.P. (2018). Polymer nanoparticles grafted zinc-containing ionic liquids: A highly efficient and recyclable catalyst for cooperative cycloaddition of CO_2_ with epoxides. J. CO_2_ Util..

[51] Chen Y.J., Luo R.C., Xu Q.H., Jiang J., Zhou X.T., Ji H.B. (2017). Charged metalloporphyrin polymers for cooperative synthesis of cyclic carbonates from CO_2_ under ambient conditions. ChemSusChem.

[52] Liu D., Li G., Liu H.O. (2018). Functionalized MIL-101 with imidazolium-based ionic liquids for the cycloaddition of CO_2_ and epoxides under mild condition. Appl. Surf. Sci..

[53] Dai W.L., Jin B., Luo S.L., Yin S.F., Luo X.B., Au C.T. (2013). Cross-linked polymer grafted with functionalized ionic liquid as reusable and efficient catalyst for the cycloaddition of carbon dioxide to epoxides. J. CO_2_ Util..

[54] Chen G.J., Zhang Y.D., Xu J.Y., Liu X.Q., Liu K., Tong M.M., Long Z.Y. (2020). Imidazolium-based ionic porous hybrid polymers with POSS-derived silanols for efficient heterogeneous catalytic CO_2_ conversion under mild conditions. Chem. Eng. J..

[55] Jiang B., Wang Y.M., Zhang L.H., Sun Y.L., Yang H.W., Wang B.Y., Yang N. (2017). Biodiesel production via transesterification of soybean oil catalyzed by superhydrophobic porous poly (ionic liquid) solid base. Energy Fuel..

[56] Pan H., Li H., Liu X.F., Zhang H., Yang K.L., Huang S., Yang S. (2016). Mesoporous polymeric solid acid as efficient catalyst for (trans) esterification of crude Jatropha curcas oil. Fuel Process. Technol..

[57] Che S.Y., Yang Z.Z., Popovs I., Luo H.M., Luo Y.L., Guo W., Chen H., Wang T., Jie K.C., Wang C.M. (2019). A succinct strategy for construction of nanoporous ionic organic networks from a pyrylium intermediate. Chem. Commun..

[58] Yi Q., Liu T.T., Wang X.B., Shan Y.Y., Li X.Y., Ding M.G., Shi L.J., Zeng H.B., Wu Y.C. (2021). One-step multiple-site integration strategy for CO_2_ capture and conversion into cyclic carbonates under atmospheric and cocatalyst/metal/solvent-free conditions. Appl. Catal. B.

[59] Li J., Jia D.G., Guo Z.J., Liu Y.G., Lyu Y., Zhou Y., Wang J. (2017). Imidazolinium based porous hypercrosslinked ionic polymers for efficient CO_2_ capture and fixation with epoxides. Green Chem..

[60] Zhang Y.D., Liu K., Wu L., Zhong H., Luo N., Zhu Y.X., Tong M.M., Long Z.Y., Chen G.J. (2019). Silanol-enriched viologen-based ionic porous hybrid polymers for efficient catalytic CO_2_ fixation into cyclic carbonates under mild conditions. ACS Sustain. Chem. Eng..

[61] Park K., Lee K., Kim H., Ganesan V., Cho K., Jeong S.K., Yoon S. (2017). Preparation of covalent triazine frameworks with imidazolium cations embedded in basic sites and their application for CO_2_ capture. J. Mater. Chem. A.

[62] Wu X., Chen C.T., Guo Z.Y., North M., Whitwood A.C. (2019). Metal- and halide-free catalyst for the synthesis of cyclic carbonates from epoxides and carbon dioxide. ACS Catal..

[63] Chaugule A.A., Tamboli A.H., Kim H. (2017). Ionic liquid as a catalyst for utilization of carbon dioxide to production of linear and cyclic carbonat. Fuel.

[64] Wilhelm M.E., Anthofer M.H., Cokoja M., Markovits I.E., Herrmann W.A., Kühn F.E. (2014). Cycloaddition of carbon dioxide and epoxides using pentaerythritol and halides as dual catalyst system. ChemSusChem.

[65] Dong T.F., Zheng Y.J., Yang G.W., Zhang Y.Y., Li B., Wu G.P. (2020). Crosslinked resin-supported bifunctional organocatalyst for conversion of CO_2_ into cyclic carbonates. ChemSusChem.

[66] Wang X.C., Zhou Y., Guo Z.J., Chen G.J., Li J., Shi Y.M., Liu Y.Q., Wang J. (2015). Heterogeneous conversion of CO_2_ into cyclic carbonates at ambient pressure catalyzed by ionothermal-derived meso-macroporous hierarchical poly(ionic liquid)s. Chem. Sci..

[67] Liu M.S., Zhao P.H., Gu Y.Q., Ping R., Gao J., Liu F.S. (2020). Squaramide functionalized ionic liquids with well-designed structures: Highly-active and recyclable catalyst platform for promoting cycloaddition of CO_2_ to epoxides. J. CO_2_ Util..

[68] Zhao Y.L., Huang H.L., Zhu H.J., Zhong C.L. (2022). Design and synthesis of novel pyridine-rich cationic covalent triazine framework for CO_2_ capture and conversion. Microp. Mesop. Mater..

[69] Zhong H., Gao J.W., Sa R.J., Yang S.L., Wu Z.C., Wang R.H. (2020). Carbon dioxide conversion upgraded by host-guest cooperation between nitrogen-rich covalent organic framework and imidazolium-based ionic polymer. ChemSusChem.

[70] Shi Z.J., Su Q., Ying T., Tan X., Deng L.L., Dong L., Cheng W.G. (2020). Ionic liquids with multiple active sites supported by SBA-15 for catalyzing conversion of CO_2_ into cyclic carbonates. J. CO_2_ Util..

[71] Fedorova I.V., Safonova L.P. (2018). Ab initio investigation of the interionic interactions in triethylammonium-based protic ionic liquids: The role of anions in the formation of ion pair and hydrogen bonded structure. J. Mater. Chem. A.

